# Direct-to-Consumer Genetic Testing Data Privacy: Key Concerns and Recommendations Based on Consumer Perspectives

**DOI:** 10.3390/jpm9020025

**Published:** 2019-05-09

**Authors:** Rachele M. Hendricks-Sturrup, Christine Y. Lu

**Affiliations:** Department of Population Medicine, Harvard Pilgrim Health Care Institute and Harvard Medical School, Boston, MA 02215, USA; christine_lu@harvardpilgrim.org

**Keywords:** genetic testing, direct-to-consumer, privacy, genetic information, genomic medicine, consumer protections

## Abstract

Direct-to-consumer genetic testing (DTC-GT) companies are engaging health consumers in unprecedented ways and leveraging the genetic information they collect to further engage health companies. This has produced controversy about DTC-GT consumer expectations, standards, and perceptions of privacy. In this commentary, we highlight recent events involving DTC-GT companies and controversy about privacy that followed those events and discuss recent studies that have explored DTC-GT consumer concerns about privacy. We discuss DTC-GT company standards of upholding consumer privacy and the general accessibility of DTC-GT company terms of use agreements and privacy policies that are written at reading levels above that of many consumers. We conclude that broader discussions and more research are needed to identify DTC-GT consumer concerns about and expectations of privacy. We anticipate that our recommendations will advance discussions on consumer privacy expectations and protections in an era of increasing engagement in DTC-GT.

Direct-to-consumer genetic testing (DTC-GT) companies provide consumers with information about ancestry, health risks, and other genetic predispositions and are engaging in the health care market in unprecedented ways. Next-generation sequencing has ushered in this new market for individuals who are eager to understand how their genetics influence their likelihood of manifesting a diverse array of phenotypes. From a health care professional perspective, however, DTC-GT is concerning; one study shows that the mere provision of genetic health risk information without a health care intermediary can lead to potential psychological effects in DTC-GT consumers [1]. From a broader perspective, a systematic literature review showed that primary care physicians are skeptical of DTC-GT and concerned about the ethical, legal, and social implications of genetic testing, namely, privacy and confidentiality issues [2]. In this commentary, we highlight recent controversy about DTC-GT company activities and studies that have explored consumers’ key concerns about DTC-GT data privacy. We discuss major challenges to upholding DTC-GT consumer expectations of privacy as a form of protection and provide recommendations.

## 1. Controversy over DTC-GT Company Activities

Using the latest advancements in next-generation sequencing technology, DTC-GT companies not only provide consumers with genealogy or ancestry services; DTC-GT companies have seamlessly entered the health care market to give consumers health reports that interpret or infer disease risks or predispositions based on the consumers’ genetic information. Over the last 10 years, policy makers and regulators, such as the United States Food and Drug Administration (FDA), have struggled to establish timely regulation of and oversight over the DTC-GT health market. An example of this struggle was seen in 2013 when the FDA warned DTC-GT company 23andMe to scale back a massive marketing campaign which sought to promote 23andMe’s genetic health risk testing services without prior FDA approval [3]. After corresponding with the FDA, 23andMe since gained the FDA’s approval to sell its genetic health risk assessment products in the market. 

Public controversy or mistrust in DTC-GT companies has surfaced and continues to resurface because DTC-GT companies are demonstrating their ability and willingness to exchange consumer information (e.g., statistics about or raw genetic health risk data and ancestry/genealogical data) with third parties [4,5,6,7]. For example, media controversy about consumer privacy followed a three-year collaboration that ended in 2018 between DTC-GT company Ancestry and Calico, a Google spinoff company [4,8,9]. This collaboration granted Calico access to Ancestry’s databases, tools, and algorithms to analyze and investigate the role and influence of genetics across families that experience unusual longevity [10,11,12]. According to its website, Ancestry’s databases currently contain over 10 million genetic information records. Media controversy about consumer privacy also followed GlaxoSmithKline in 2018 when it announced that it would leverage information from 23andMe’s databases to identify and select pharmaceutical targets [7,13,14,15,16]. 

## 2. DTC-GT Consumer Comprehension of Terms of Use Agreements and Privacy Policies

In 2017, consumer protection attorney and former New Jersey deputy and Department of Justice Attorney General Joel Winston gave a written warning to Ancestry customers. The warning stated that although consumer ownership over Ancestry DNA is limited to years, Ancestry owns the consumers’ DNA in perpetuity [17]. Winston further discussed Ancestry’s Privacy Policy and Terms of Use agreements, and encouraged consumers to consider three significant provisions: “(1) the perpetual, royalty-free, worldwide license to use your DNA; (2) the warning that DNA information may be used against ‘you or a genetic relative’; and (3) your waiver of legal rights” [17]. Terms of use agreements and privacy policies are sometimes referred to as “click-wrap” or “browse-wrap” agreements. These agreements are concerning because, although they are time-convenient, they are sometimes written at college reading levels that can be difficult for some consumers to read and comprehend [18,19,20,21]. It is therefore questionable as to whether consumers fully or partially understand DTC-GT company consumer policies and agreements even when consumers take time to read them [18,19,20,21]. 

Consumer perceptions of genetic information privacy vary depending on how well DTC-GT consumers read and comprehend privacy policies and terms of use agreements [22]. The United States National Library of Medicine (US NLM) understands how this variation may exist and has provided formal recommendations to address it. Specifically, the US NLM recommends that written materials published or provided by companies who offer consumer health information should be written at seventh- to eighth-grade reading levels [23]. 

## 3. Consumer Concerns and Perceptions about DTC-GT Information Privacy 

During recent years, researchers have explored DTC-GT consumer concerns and perceptions about genetic information privacy. Key findings from some of their studies are summarized in Table 1. Key consumer concerns appear to center on governmental oversight and regulation of DTC-GT company activities, third-party access to DTC-GT information, consumer motivations to participate in the sharing or exchange of genetic information, and consumer expectations of privacy after publicly sharing personal genetic health information. Consumer perceptions of benefits, harms, and limitations to engagement in DTC-GT, and trust in regulation and upholding of privacy, are also presented in Table 1.

Results from these studies show that consumers may engage in DTC-GT for various personal reasons, which include but are not limited to satisfying their desire to learn about their genetic health risks. Simultaneously, consumers have concerns about or value the importance of maintaining their privacy and upholding regulations that protect their privacy. 

Critchley et al. discovered, among the consumer concerns they identified, that the least trusted aspects of DTC-GT companies related to matters of consumer privacy. Adding to this finding is that of Haeusermann et al., who found that some DTC-GT consumers perceived privacy is an “illusion” [22]. This perception was based on the notion that electronic genetic information files can be easily hacked [22]. This particular concern lends true or can be substantiated by recent events involving electronic health record breaches, which have affected over half of the United States’ (US) population [29,30]. 

Concerns were also raised about physicians’ use of consumer health information in Critchley et al.’s study [28]. Although physicians are presumed to be gatekeepers of private health information, consumers felt that physicians might use patient information for promotional purposes without patient consent [28]. Findings from Critchley et al.’s and Haeusermann et al’s. studies show that some consumers may not trust that their DTC-GT information is private when the information is controlled by a physician or stored within electronic health records. 

Gollust et al. discovered that although over half (62.9%) of DTC-GT consumers in their sample felt that genetic information should be included in the standard medical record, although the majority of consumers (89.9%) in their sample also felt it necessary to maintain personal control over the use or exchange of their genetic health information without a clinician gatekeeper [26]. Only a small portion (14.3%) of the participants felt that access to DTC-GT should occur only through a doctor [26]. Consumer preferences to reduce or eliminate clinician control over accessing and exchanging genetic health information, via consumers’ direct engagement with DTC-GT companies, challenge the perceived role of the clinician as the primary health information keeper or consultant for patients. 

## 4. Meeting Health Care Consumer Standards and Preferences of Privacy—What We Recommend 

The Future of Privacy Forum, a Washington DC-based think tank and advocacy group, met with leading DTC-GT companies (23andMe, Ancestry, Helix, MyHeritage, and Habit) to establish best “voluntary” practices for genetic information use and security [31]. They concluded that the best consumer privacy practices should (1) promote transparency about how consumer genetic information is used, collected and shared; (2) provide consumers with choices about consent for participation in research and destruction of their DNA samples; and (3) enhance consumer protections to ensure that their genetic information is shared in accordance with applicable laws and with the utmost discretion (e.g., strong data security practices and valid legal process requirements for disclosure) [31]. 

Considering the research findings presented herein, we conclude that patients, as consumers and autonomous partners in their own health care, want personal control over and convenient access to their genetic health records. We thus advocate for an additional best practice among those presented by the Future of Privacy Forum: DTC-GT company terms of use agreements and privacy policies should be written at reading levels recommended by the US NLM to better inform consumers. This includes stricter regulatory oversight over DTC-GT companies to ensure that their terms of use agreements and privacy policies are written at the recommended reading level, which we believe adds to our recommendation for enhanced consumer privacy protection through ethically sound and enforceable laws, regulations, and policies [32].

In addition, broader discussions are needed to determine if click-wrap or browse-wrap terms of use agreements and privacy policies are appropriate in circumstances involving the exchange of potentially actionable or clinically relevant genetic health data with DTC-GT consumers. The convenience of click-wrap or browse-wrap agreements and policies could potentially undermine the value of actionable or clinically relevant genetic health information. 

Given Critchley et al.’s findings, which suggested there are consumer concerns about clinicians exchanging patient health information in the health care market without patient consent, further research is needed to assess and define health consumer trust in health care providers. This is especially important because the provision of health care services is market-driven in many countries like the US.

In summary, we have discussed public controversy over DTC-GT company activity, how consumer expectations of privacy can be misguided by language in terms of use agreements and privacy policies, and DTC-GT consumer perceptions of and desire to uphold their privacy. We anticipate that our recommendations will advance discussions of DTC-GT consumer privacy in an era of increasing use of DTC-GT. 

## Figures and Tables

**Table 1:** Direct-to-consumer genetic testing (DTC-GT) consumer perceptions about genetic information privacy [22,24,25,26,27,28].

Author(s), Year	Population Surveyed or Interviewed	Perceptions and Motivations Identified
**Bollinger et al., 2013**	DTC-GT consumers living in the US (Navigenics, 23andMe, and deCODEme; *n* = 1046)	Perceptions about governmental oversight and third-party access to consumers’ genetic information: Oversight is either very important (45%) or somewhat important (39%). Having a governmental agency, like the Federal Trade Commission, monitor the claims made by DTC companies is very (34%) or somewhat (39%) important. It is very (36%) or somewhat important (30%) that services provided by DTC companies be available without governmental oversight. It is very (87%) or somewhat (9%) important that it be illegal for insurers and employers to get DTC genetic information. It was very (74%) or somewhat (15%) important that it be illegal for law enforcement to get DTC genetic information.
**Haeusermann et al., 2017**	Public genealogy database users (openSNP users; majority reported from the US (60.33%), Canada (5.17%), and United Kingdom (4.61%); *n* = 550)	Motivation to participate in genetic information sharing: Learn more about oneself. Contribute to the advancement of medical research. Help improve the predictability of genetic testing. Consider it fun to explore genotype and phenotype data.
**Haeusermann et al., 2018**	Public genealogy database users (openSNP users from the United States, Canada, United Kingdom, Australia, Switzerland, and Russia; *n* = 13)	Understandings of privacy based on experience in engaging in a public genealogy database: Publicly sharing genomic data affects the individual and has potential consequences for family members and future generations (could unveil significant information about a family’s entire health and genealogical history). Skepticism among family members about publicly sharing genetic information. Privacy can be easily breached, regardless of the intent for sharing genetic information. Privacy is an “illusion”; hackers can easily gain access to any kind of information, even institutional, such as government files. Protecting privacy is an impossible task. Prior engagement with third parties due to special life circumstances already granted medical, insurance, legal, and governmental institutions access to individuals’ data (prior loss of control over one’s genetic information). Privacy risks don’t affect me directly (I do not belong to vulnerable social groups (e.g., ethnic or sexual minorities), that are more exposed to discrimination). Reducing privacy risks for minority groups is key to fostering scientific progress and should be a government priority. Vulnerable groups should receive special attention because they might perceive themselves as at a higher risk for discrimination. Opt-out policies (i.e., sharing data by default) would increase data sharing.
**Gollust et al., 2017**	DTC-GT consumers (23andMe; *n* = 941)	Experiences with personal genome testing (PGT) and the impact of those experiences on consumer views about the availability and regulation of PGT: 89.9% felt individuals should have a right to access their own genetic information without a gatekeeping medical professional. 83.2% of consumers felt it important that genetic information be kept private. 62.9% felt that genetic information should be included in the standard medical record. 60.3% felt that insurance should cover the cost of personal genomic testing. 27.8% felt that more government effort is needed to regulate personal genomic testing. 14.3% felt that access to DTC-GT should occur only through a doctor.
**Roberts et al., 2017**	DTC-GT consumers (23andMe and Pathway Genomics; *n* = 1648)	Perceived benefits, harms, and limitations in DTC-PGT after undergoing testing: 20.8% did “not at all” consider genetic privacy (20.8%) in their pursuits to obtain DTC-PGT.
**Critchley et al., 2015**	Australian adults over the age of 18 years within the 2012 Swinburne National Technology and Society Monitor (*n* = 1000)	Feelings of trust in regulation and upholding of privacy (DTC-GT company (*n* = 489) versus general practitioner (GP; *n* = 511)): Significantly more likely to trust the regulation and privacy associated with a genetic test provided by a GP compared with a DTC company; least trusted aspects associated with DTC-GT companies related to privacy. Privacy was one of the most trusted aspects associated with genetic testing via a GP. Consumers suspect that GPs may use patient information for promotion purposes without patient consent.
